# Importance of routine alcohol screening and brief intervention in women

**DOI:** 10.1186/1940-0640-7-S1-A48

**Published:** 2012-10-09

**Authors:** Aruna Chhabria, J Paul Seale

**Affiliations:** 1 Department of Family Medicine, Mercer University School of Medicine, Macon, GA, USA

## 

We present a case report and review of the literature to demonstrate why screening, brief intervention, and referral to treatment (SBIRT) is important for female primary-care patients. A 25-year-old African American woman presented with right lower-quadrant and flank pain, nausea, fever, and dyspareunia three days after unprotected intercourse. The patient had a past history of sexually transmitted infections. An initial alcohol screening was negative for heavy episodic drinking. Physical exam revealed fever, tachycardia, costovertebral angle tenderness, pelvic tenderness, vaginal discharge, and cervical motion tenderness. Urine analysis showed pyuria and bacteriuria. The patient was admitted with pelvic inflammatory disease (PID) and pyelonephritis. Repeat screening by an SBIRT-trained resident revealed recurrent weekend heavy episodic drinking with unprotected intercourse. The patient was treated with antibiotics and brief intervention. During the intervention, she demonstrated awareness of the link between heavy episodic drinking and PID. She reported high level of importance and readiness to decrease her drinking to prevent recurrences. She improved quickly and was discharged on hospital day three but failed to follow up. This case supports the following findings from the research literature: significant high rates of risky drinking among women, the link between alcohol consumption and high-risk health behaviors, the hesitancy of many women to disclose alcohol misuse, and the importance of linking alcohol misuse identified through brief intervention in primary care with alcohol-related consequences. Routine alcohol screening is indicated for all adult patients, and SBIRT training is an important element of primary-care residency training. Nonjudgmental alcohol screening identified binge drinking in a patient who previously denied it, helped the patient recognize the link between alcohol misuse and hospitalization, and enhanced the patient’s motivation to change. Effective SBIRT in such situations have the potential to prevent recurrent alcohol-related hospitalizations among at-risk drinkers.

